# Ex vivo model of epilepsy in organotypic slices—a new tool for drug screening

**DOI:** 10.1186/s12974-018-1225-2

**Published:** 2018-07-11

**Authors:** Daniela M. Magalhães, Noémia Pereira, Diogo M. Rombo, Cláudia Beltrão-Cavacas, Ana M. Sebastião, Cláudia A. Valente

**Affiliations:** 1 Instituto de Farmacologia e Neurociências, Faculdade de Medicina, Universidade de Lisboa, Lisboa, Portugal; 2 Instituto de Medicina Molecular, Faculdade de Medicina, Universidade de Lisboa, Lisboa, Portugal

**Keywords:** Epilepsy, Organotypic slice cultures, Neuroinflammation, Gliosis, Proinflammatory cytokines, Interleukin-1β, NLRP3 inflammasome

## Abstract

**Background:**

Epilepsy is a prevalent neurological disorder worldwide. It is characterized by an enduring predisposition to generate seizures and its development is accompanied by alterations in many cellular processes. Organotypic slice cultures represent a multicellular environment with the potential to assess biological mechanisms, and they are used as a starting point for refining molecules for in vivo studies. Here, we investigated organotypic slice cultures as a model of epilepsy.

**Methods:**

We assessed, by electrophysiological recordings, the spontaneous activity of organotypic slices maintained under different culture protocols. Moreover, we evaluated, through molecular-based approaches, neurogenesis, neuronal death, gliosis, expression of proinflammatory cytokines, and activation of NLRP3 inflammasome (nucleotide-binding, leucine-rich repeat, pyrin domain) as biomarkers of neuroinflammation.

**Results:**

We demonstrated that organotypic slices, maintained under a serum deprivation culture protocol, develop epileptic-like activity. Furthermore, throughout a comparative study with slices that do not depict any epileptiform activity, slices with epileptiform activity were found to display significant differences in terms of inflammation-related features, such as (1) increased neuronal death, with higher incidence in CA1 pyramidal neurons of the hippocampus; (2) activation of astrocytes and microglia, assessed through western blot and immunohistochemistry; (3) upregulation of proinflammatory cytokines, specifically interleukin-1β (IL-1β), interleukin-6, and tumor necrosis factor α, revealed by qPCR; and (4) enhanced expression of NLRP3, assessed by western blot, together with increased NLRP3 activation, showed by IL-1β quantification.

**Conclusions:**

Thus, organotypic slice cultures gradually deprived of serum mimic the epileptic-like activity, as well as the inflammatory events associated with in vivo epilepsy. This system can be considered a new tool to explore the interplay between neuroinflammation and epilepsy and to screen potential drug candidates, within the inflammatory cascades, to reduce/halt epileptogenesis.

**Electronic supplementary material:**

The online version of this article (10.1186/s12974-018-1225-2) contains supplementary material, which is available to authorized users.

## Background

Epilepsy is among the most prevalent neurological disorders worldwide. According to World Health Organization, epilepsy accounts for 1% of global burden of disease [1].

Clinically, epilepsy is characterized by recurrent spontaneous seizures and is defined, by the International League Against Epilepsy (ILAE), as “a chronic condition of the brain characterized by an enduring propensity to generate epileptic seizures, and by the neurobiological, cognitive, psychological, and social consequences” [2]. Despite the numerous antiepileptic drugs (AEDs) available, 30% of patients are still refractory to therapy, continue to experience seizures, and a subset suffer progression of the disease, with increasing seizure frequency and cognitive decline. It is therefore imperative to better understand the mechanisms of epileptogenesis, the process by which a normal brain transforms into one capable of producing recurrent spontaneous seizures, and to find novel and specific therapies to prevent/delay the onset of this disorder, as well as to ameliorate its symptoms, and ultimately to cease its progression.

Epileptogenesis is accompanied by several cellular and molecular processes, such as neuronal death, neurogenesis, reactive astrogliosis and microglia activation, and upregulation of inflammatory mediators [3].

The effect of seizures on neuronal death and the role of seizure-induced neuronal death in epileptogenesis have been intensely discussed [4]. αII-Spectrin, a structural protein of the cell cytoskeleton with a molecular weight of 250 kDa, is a major substrate of cytosolic cysteine proteases, such as calpains and caspases [5]. Calpains are the main proteases involved in necrosis, while the effector caspase-3 is considered essential for efficient execution of apoptosis [6, 7]. Neuronal death leads to activation of these cysteine proteases, αII-spectrin cleavage, and formation of spectrin breakdown products (SBDP). Since αII-spectrin occurrence in glial cells is minimal, SBDPs are considered highly specific for neuronal damaged, as well as biomarkers of cell death in brain disorders [8, 9].

Prolonged seizure activity leads to a dramatic increase in cell proliferation in the dentate gyrus (DG) [10, 11] and rostral subventricular zone [12]. In the DG, the accelerated neural stem cell proliferation is reflected by an increased number of cells expressing doublecortin (DCX) [11], a validated marker for newborn neurons [13]. However, the role of new neurons, and their migration, in the epileptic process is still under debate [14].

Non-neuronal cells, especially astrocytes and microglia, play a prominent role in the pathophysiology of epilepsy [15]. Astrocytes are the third element of the tripartite synapse [16–18] and active players in neuroinflammation [19]. They respond to CNS insults by a process commonly referred to as reactive astrogliosis, a graded continuum of progressive alterations [20, 21]. Microglia cells are the main sensors for pathological events in the CNS, and evidence indicates that the unregulated activation of microglia in response to noxious stimulus results in the production of toxic factors that propagate neuronal injury [22, 23]. Moreover, as happens with astrocytes, microglia activation occurs as a graded response with microglia adopting different morphologies, ranging from a highly ramified to an amoeboid-like phenotype [24–26].

Experimental and clinical evidences have also demonstrated that seizures induce high levels of inflammatory mediators in brain regions involved in the generation and propagation of epileptic activity and proinflammatory cytokines, such as interleukin-1β (IL-1β), tumor necrosis factor-α (TNF-α), and interleukin-6 (IL-6), which were found to be upregulated in activated microglia and astrocytes [27]. Recently, NOD-like receptor protein 3 (NLRP3) inflammasome has been associated to central nervous system (CNS)-related disorders [28–30]. NLRP3 is a cytosolic multiprotein complex that assembles in response to invading pathogens (PAMPs, pathogen-associated molecular patterns) and danger signals (DAMPs, damaged-associated molecular patterns), which results in the processing of inactive pro-caspase-1 into the active cysteine-protease enzyme caspase-1, that subsequently processes the pro-IL-1β and pro-IL-18, prompting the production of mature IL-1β and IL-18 and inducing pyroptosis, a highly pyrogenic inflammatory form of cell death. Research suggests that NLRP3-targeted therapies may represent a novel antiepileptogenic strategy [31]. Indeed, NLRP3 inhibition was already reported to provide neuroprotection in a kindling model of epilepsy [32].

Epileptogenesis is difficult to study in humans, due to the heterogeneity of epileptogenic injuries, long latent periods lasting months to decades, and the potentially confusing effects of anticonvulsant treatment after the first spontaneous seizure. Animal models of epilepsy have been crucial in the understanding of the physiological and behavioral changes associated with human epilepsy and they have led to the discovery of many AEDs [33, 34], but they require a large number of animals and data collection is quite slow due to the long latent period until seizures onset [35].

Organotypic hippocampal slice cultures (OHSC), which are prepared from slices of explanted tissue, represent a complex multicellular ex vivo environment and constitute a powerful instrument to elucidate biological mechanisms [36, 37]. These cultures preserve the three-dimensional architecture and local environment of brain cells, including neurons, astrocytes, and microglia, as well as the neuronal connectivity and the complex glial-neuronal interactions. Furthermore, cells in organotypic brain slices develop and mature similarly to their in vivo counterparts [38]. Nowadays, these systems are considered excellent models to study neuroprotection and they are used as a starting point for drug discovery [39, 40]. Ex vivo models of epilepsy in hippocampal slices allow a detailed and well-controlled research of the mechanisms of epileptogenesis, while still preserving the network phenotypic features of epilepsy, particularly the development of spontaneous seizures [41]. Indeed, spontaneous field potential activity was recorded from organotypic slices [42, 43]. It was reported that slices develop epileptiform activity which resembles in vivo epilepsy, including sensitivity to anticonvulsants and increasing seizure incidence over time. Detailed characterization of this system in terms of spontaneous interictal and ictal-like events allowed to present it as a useful model system for investigating the mechanisms of epileptogenesis, as well as to screen potential antiepileptic drugs [42, 44].

In this study, we aimed to further explore organotypic slices as a model of epilepsy. We confirmed the occurrence of spontaneous epileptiform activity in slices maintained in Neurobasal/B27 serum-free medium, and showed the strong incidence of neuroinflammation biomarkers in these slices, as the enhanced expression and activation of NLRP3 inflammasome.

We thus demonstrate that organotypic slice cultures gradually deprived of serum mimic the epileptic-like activity, as well as many inflammatory events associated with in vivo epilepsy. Studies within this system can contribute to a deeper knowledge about the interplay between epilepsy and inflammation. Furthermore, this model can be used as a tool to screen potential therapeutic candidates for epilepsy.

## Methods

### Animals

Pregnant female Sprague-Dawley rats were acquired from Charles River (Barcelona, Spain). The Portuguese law and European Union guidelines (2010/63/EU) were respected in all procedures regarding the protection of animals for scientific purposes. All efforts were made to minimize animal suffering and to use the minimum number of animals. This study was approved by the “iMM’s Institutional Animal Welfare Body–ORBEA-iMM and the National competent authority–DGAV (Direção Geral de Alimentação e Veterinária).”

### Organotypic slice cultures

**Fig. 1:**
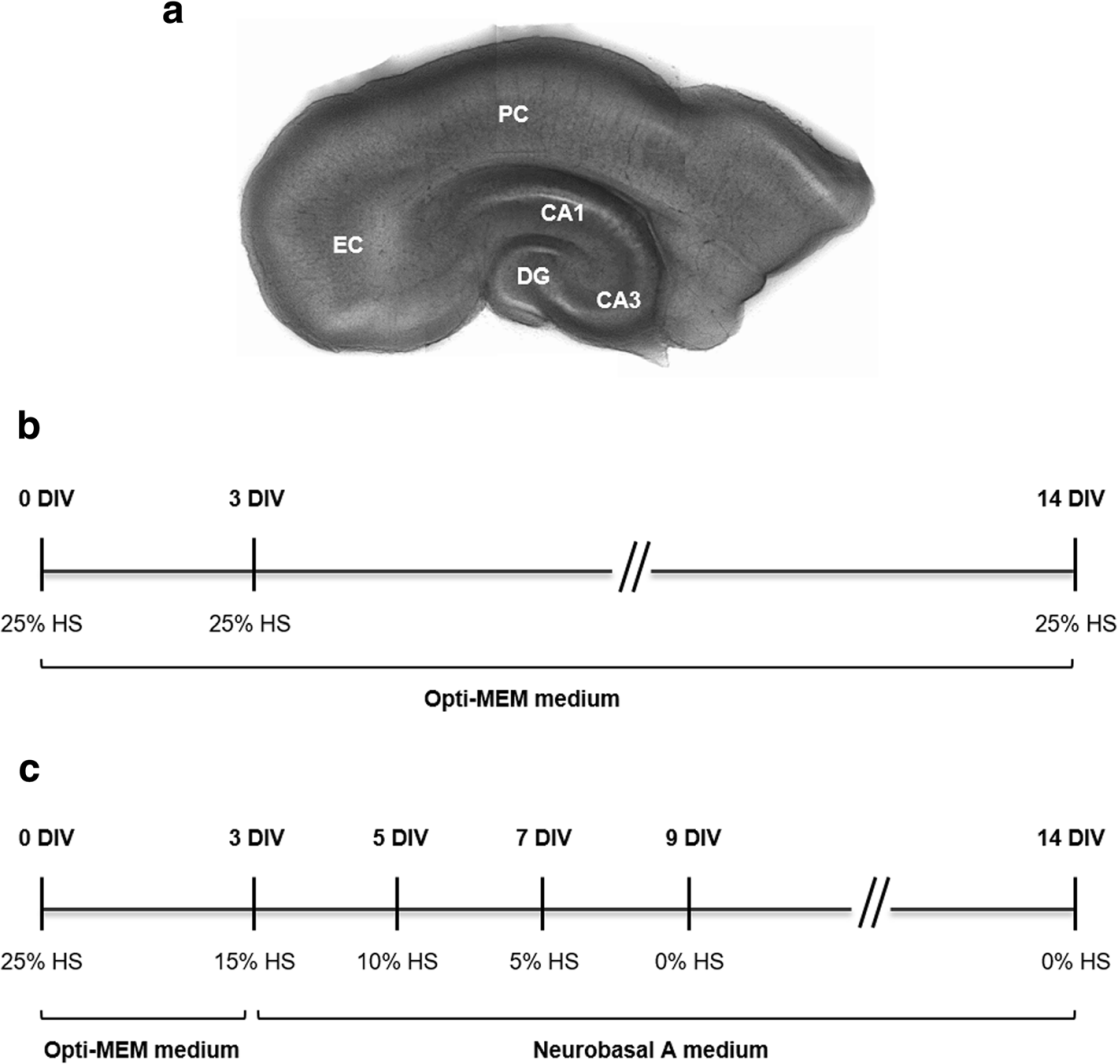
Representative photograph of a combined entorhinal cortex-hippocampus slice immediately after dissection. **a** All slices include the cornu ammonis (CA), dentate gyrus (DG), entorhinal cortex (EC), and perirhinal cortex (PC). **b**, **c** Schematic representation of culture maintenance protocol for organotypic slices control (CTL) and with spontaneous epileptiform activity (EL). **b** CTL slices were grown in Opti-MEM-based medium supplemented with 25% horse serum (HS). Medium renewal, starting at 3 days in vitro (DIV), was performed twice a week. **c** In EL slices, at 3 DIV, the medium was changed to Neurobasal A-based medium in the presence of decreasing concentrations of HS (15, 10, and 5%) until complete serum-free medium was reached at 9 DIV. Medium renewal, starting at 3 DIV, was performed every second day

Organotypic slice cultures were prepared from 6- to 7-day-old Sprague-Dawley rats, according to the interface culture method [45]. After decapitation, brains were removed and placed in cold Gey’s balanced salt solution (GBSS, Biological Industries, Kibbutz Beit Haemek, Israel) with 25 mM d-glucose (Sigma, St. Louis, MO, USA), under sterile conditions. The hippocampus, together with the entorhinal cortex (EC) and perirhinal cortex (PC), (Fig. 1a), was dissected out and sliced transversely at 350 μm using a McIlwain tissue chopper. Five slices were placed onto porous (0.4 μm) insert membranes (PICM 03050, Millipore, Bedford, MA), which were transferred to six-well culture trays (Corning Costar, Corning, NY). Each well contained 1 ml of culture medium composed of 50% Opti-MEM, 25% Hanks’ balanced salt solution (HBSS), 25% heat-inactivated horse serum (HS) (all from Invitrogen, Paisley, UK), 25 mM d-glucose, penicillin (100 units/ml), and streptomycin (100 μg/ml) (Sigma). Slices were maintained at 37 °C with 5% CO_2_ and 95% atmospheric air for the following 2 weeks.

Slices were randomly divided in two groups, which undertook different culture conditions (Fig. 1). Those designated control slices (CTL, Fig. 1b) were kept in a serum-based (25% HS) Opti-MEM medium, with medium renewal twice a week, and did not develop epileptiform activity. Those slices which spontaneously develop epileptiform activity (EL, Fig. 1c) were changed at 3 days in vitro (DIV) to a chemically defined serum-free based medium, Neurobasal A (Invitrogen), supplemented with 2% B27 (Invitrogen), l-glutamine (1 mM) (Invitrogen), penicillin (100 units/ml), and streptomycin (100 μg/ml), and decreasing HS concentrations (15%, 10% and 5%), until a serum-free condition was reached at 9 DIV. In EL slices, medium was renewed every second day. Our protocol is different from other reports in the sense that slices are gradually deprived of serum.

### Field potential recordings

Field potential recordings were performed in an interface-type chamber as previously described by others [20]. At 14 DIV, slices were transferred to the interface recording chamber, with a humidified (5% CO_2_/95% O_2_) atmosphere at 36 °C, and with their growth medium continuously superfused and recirculating at a rate of 2 ml/min. Each slice was visually inspected, ensuring slice integrity and organization. After an equilibration period of 20 min, spontaneous field potential recordings were performed with an extracellular microelectrode (4 M NaCl, 2–4 MΩ resistance) positioned in the CA3 pyramidal cell layer over 30–40 min. Recordings were obtained with Axoclamp 2B amplifier, digitized (Axon Instruments, Foster City, CA), and analyzed by the Clampex software version 10.2 (Molecular Devices, Sunnyvale, CA, USA). All recordings were band-pass filtered (eight-pole Bessel filter at 60 Hz and Gaussian filter at 600 Hz).

### Patch-clamp recordings

Slices were transferred to an Axioskop 2FS upright microscope (Zeiss, Jena, Germany) equipped with a differential interference contrast-infrared (DIC-IR) CCD video camera (VX44, Till Photonics, Gräfelfing, Germany) and fixed with a grid in a recording chamber continuously superfused by a gravitational superfusion system at 2–3 ml/min with artificial cerebrospinal fluid (aCSF) containing (in mM) NaCl 124, KCl 3, NaH_2_PO_4_ 1.25, NaHCO_3_ 26, MgCl_2_ 1, CaCl_2_ 2, Glucose 10, pH 7.4 (gassed with 95% O_2_, 5% CO_2_), at room temperature. Patch pipettes (4–9 MΩ) were pulled from borosilicate glass capillaries (1.5 mm outer diameter, 0.86 mm inner diameter, Harvard Apparatus, Holliston, MA, USA) with PC-10 Puller (Narishige Group, London, UK) and were filled with an internal solution containing (in mM) K-gluconate 125, KCl 11, CaCl_2_ 0.1, MgCl_2_ 2, EGTA 1, HEPES 10, MgATP 2, NaGTP 0.3 and phosphocreatine 10, pH 7.3 adjusted with KOH (1 M), 280–290 mOsm. Recordings were performed in current-clamp mode using an EPC-7 electrical amplifier (List Biologic). Signals were low-pass filtered using a 3- and 10-kHz three-pole Bessel filter of an EPC-7 amplifier, digitized at 10 kHz using a Digidata 1322A board, and registered by the Clampex software version 10.2 (Molecular Devices). Resting membrane potential (RMP) was measured immediately after establishing whole-cell configuration, and action potential firing was systematically evoked in current-clamp mode by injecting current pulses of − 50 to + 300 pA, in 10 or 50 pA increments for 1000 ms from an initial holding potential of − 70 mV. The threshold for action potential (AP) generation was determined as the difference between the resting membrane potential and the membrane potential at which phase plot slope reached 10 mV/ms [46].

### Western blot analysis

Hippocampi were dissected from five slices and dissociated by passing 20 times through a 25G needle (Terumo Europe, Leuven, Belgium) in 150 μL of ristocetin-induced platelet agglutination buffer (RIPA, 50 mM Tris pH 8.0, 1 mM EDTA (ethykenediamine tetraacetic acid), 150 mM NaCl, 1% NP40 substitute (Nonyl phenoxlpoylethanol, Fluka Biochemika, Switzerland), and 10% glycerol), containing a mixture of protease inhibitors (Sigma). Dissociated tissue was shaken for 15 min at 4 °C. Tissue lysates were then centrifuged at 13000×*g* for 10 min to remove cell debris. The supernatant was collected, and total protein was quantified using the Bio-Rad DC Protein Assay Kit (Bio-Rad, Hercules, CA, USA).

Samples (35 μg total protein/well) and protein molecular weight marker (NZYColour Protein Marker II, NZYTech, Lisbon, Portugal) were boiled at 95 °C for 10 min, electrophoresed on a 12% SDS-PAGE, and electrotransferred to PVDF membranes (Merck-Millipore, Feltham, UK). Following blocking, membranes were probed with the primary antibodies and with the appropriate horseradish peroxidase (HRP)-conjugated secondary antibodies (1:10000, Santa Cruz Biotechnology, Heidelberg, Germany) as previously described [47]. Immunoreactions were visualized with ECL Western Blotting Detection System (GE Healthcare, Buckinghamshire, UK). The integrated intensity of each band was calculated using computer-assisted densitometry analysis with ImageJ software 1.44b. Glyceraldehyde-3-phosphate dehydrogenase (GAPDH) was used as the loading control.

Images were prepared for printing in Image Lab software 5.2.1 (software available in ChemiDoc XRS+ system, Bio-Rad). For each protein evaluated, the chemiluminescence image was merged with the colorimetric image of the molecular weight marker.

The primary antibodies used were mouse monoclonal antibody anti-αII-spectrin (1:500) and rabbit polyclonal antibody anti-Caspase-3 (1:1000) from Santa Cruz Biotechnology; rabbit polyclonal antibody anti-GFAP (1:5000) from Sigma; and goat polyclonal antibody anti-Iba1 (1:1000), rabbit polyclonal antibody anti-NLRP3 (1:300), and mouse monoclonal antibody anti-GAPDH (1:5000), all from Abcam, Cambridge, UK.

### Quantitative real-time PCR

Hippocampi were isolated from ten slices, and RNA was extracted according to QIAGEN RNeasy Mini Kit (QIAGEN, Hilden, Germany). The collected tissue was dissociated with a 25G needle in the presence of QIAzol lysis reagent. RNA concentration was determined using Nanodrop 1000 (ND-1000 Spectrophotometer, Thermo Fisher Scientific, Waltham, MA, USA).

In vitro reverse transcription was performed from 2 μg of total RNA (in 20 μl) and carried out with SuperScript II Reverse Transcriptase (EC 2.7.7.49, Invitrogen, Carlsband, CA, USA) in a thermoclycler (MyCycle, Bio-Rad), according to the manufacturer’s recommendations (SuperScript First Strand Synthesis Systems for RT-PCR, Invitrogen). cDNA amplification was carried out in a Rotor-Gene 6000 real-time rotary analyzer thermocycler (Corbett Life Science, Hilden, Germany) as former described [47]. Briefly, the reactions took place in the presence of SYBR Green Master Mix (Applied Biosystems, Foster City, CA, USA) and 0.2 μM of each gene-specific primers. RT-qPCR parameters included an initial denaturation step for 2 min at 94 °C and 50 cycles with 30 s at 94 °C, 90 s at 60 °C, and 60 s at 72 °C. Reaction specificity was evaluated in all assays by a melting curve (Additional file 1: Figure S1).

The threshold cycle (Ct) and the melting curves required for the relative quantification [48] were acquired with Rotor-Gene 6000 Software 1.7 (Corbett Life Science). GAPDH was used as the reference internal standard.

The primers used for the proinflammatory cytokines (see Table 1) were designed using the OligoAnalyzer 3.1, provided by Integrated DNA Technologies (Coralville, IA, USA) and acquired to Invitrogen. All mRNA sequences from *Rattus norvegicus* were obtained from the GenBank sequence database of the National Centre for Biotechnology Information (http://www.ncbi.nlm.nih.gov/nucleotide/). Accession numbers are indicated in Table 1.

**Table 1:** Primers used for the analysis of proinflammatory cytokines by RT-PCR. All primers were designed using the OligoAnalyzer 3.1, provided by Integrated DNA Technologies

Gene	Acession number	Primer sequence (5′–3′)	PCR fragment size (bp)
Proinflammatory cytokines			
IL-1β	NM_031512.2	Forward: TCCTCTGTGACTCGTGGGAT	309
		Reverse: GTTTGGGATCCACACTCTCCA	
TNF-α	NM_012675.3	Forward: ATGGGCTCCCTCTCATCAGT	106
		Reverse: GCTTGGTGGTTTGCTACGAC	
IL-6	NM_012589.2	Forward: GCAAGAGACTTCCAGCCAGT	203
		Reverse: TTGCCATTGCACAACTCTTTTCT	
Housekeeping gene			
GAPDH	NM_017008	Forward: GTTTGTGATGGGTGTGAACC	170
		Reverse: TCTTCTGAGTGGCAGTGATG	

The table indicates the gene, the gene accession number, the primer sequence, and the PCR fragment size

### Quantification of cell death by propidium iodide uptake

Cell death was assessed by monitoring the cellular uptake of the fluorescent dye propidium iodide (PI, 3,8-diamino-5-(3-(diethylmethylamino)propyl)-6-phenyl phenanthridinium diiodide, Sigma). PI is a polar compound, which only enters cells with damaged cell membranes and interacts with DNA emitting red fluorescence (630 nm; absorbance 493 nm). It is not toxic to cells and was proven to be a feasible marker of neuronal cell death in organotypic slice cultures [49].

PI imaging was carried out at 14 DIV. CTL and EL slices were incubated with 2 μM sterile PI, diluted in culture medium for 4 h before imaging. Cellular uptake of PI was recorded by fluorescence microscopy on a wide field fluorescence microscope (Axiovert 200, Zeiss, Germany) using a rhodamine filter. Fluorescence photomicrographs were acquired, under identical lighting, with an EC Plan-NeoFluar 5x objective (Zeiss) with a numerical aperture of 0.16. For assessment of PI uptake, the regions of interest (DG, CA1 and CA3) were delineated and the fluorescence intensity was quantified using the software ImageJ 1.44b (NIH). The intensity value of each analyzed region was obtained by correction with a fluorescence background image. PI uptake by each hippocampal region was expressed in arbitrary units of fluorescence intensity.

### Immunohistochemical analysis

Slices were fixed, at 14 DIV, for 1 h with 4% paraformaldehyde (PFA, Sigma) diluted in PBS at room temperature (RT), followed by an incubation in increasing concentrations of a sucrose (Sigma) solution (10 and 20% in PBS) at RT. Slices were kept in a 30% sucrose solution at 4 °C until further use.

Slices were cut out of the insert and put in slides. Each slice was surrounded with DAKO pen (Dako, Glostrup, Denmark) to protect staining areas from drying out and from mixing with each other. Following PBS washes, slices were incubated for 3 h at RT in blocking solution containing 10% HS, 10% bovine serum albumin (BSA, Sigma), and 1% Triton X-100 (Sigma) in PBS, which ensure simultaneous permeabilization and blocking of the tissue. Subsequently, slices were incubated with the primary antibodies for 24 h at 4 °C. Slices were rinsed with PBST (PBS containing 0.1% Tween-20), and the fluorophores coupled-secondary antibodies were applied to the slices for 4 h at RT. The nuclei were stained with Hoechst 33342 (20 μg/ml, Invitrogen) for 40 min at RT. The slices were mounted in Mowiol (Sigma).

For each marker, images were acquired under identical lighting between conditions, with a frame size of 1024 × 1024 pixels on an inverted confocal laser scanning microscope (Zeiss LSM 710, Zeiss) equipped with a Plan-Apochromat 20x objective (Zeiss) with a numerical aperture of 0.80. Hoechst 33342 fluorescence was detected with a 405-nm diode laser (30 mW nominal output). Alexa Fluor 488 fluorescence was detected using the 488-nm line of an Argon laser (25 mW nominal output), and Alexa Fluor 568 was detected using a 561-nm DPSS laser (15 mW nominal output). Images were prepared for printing using Illustrator software from Adobe Systems (San Jose, CA, USA).

Doublecortin (DCX) fluorescence intensity was quantified in the DG of CTL and EL slices, using the software ImageJ 1.44b. The final intensity value was obtained by correction with a fluorescence background image. Fluorescence intensity values are presented as a percentage of CTL slices.

The microglia cell body diameter in CTL and EL slices stained with Iba1 was measured, using the software ImageJ 1.44b, in 50–70 microglia cells per each hippocampal area. Whenever the cell body was oval, the cell body diameter was considered the largest distance passing through the center of the ellipse.

The primary antibodies used were mouse monoclonal antibody anti-GFAP (1:1000, Sigma), goat polyclonal antibody anti-Iba1 (1:1000, Abcam), and mouse anti-doublecortin (1:500, Santa Cruz Biotechnology). The secondary antibodies were donkey anti-mouse-Alexa Fluor 488, donkey anti-goat-Alexa Fluor 488, and donkey anti-mouse-Alexa Fluor 568 (1:500, Invitrogen).

### ELISA

Hippocampi were dissected from five slices and homogenized, as for western blot, in ice-cold RIPA buffer containing a mixture of protease inhibitors (Sigma). The lysates were centrifuged, and the supernatants, containing the cytosolic and membrane fraction, were collected to measure IL-1β, TNF-α, and IL-6 in the tissue. Total protein was quantified using the Bio-Rad DC Protein Assay Kit (Bio-Rad). IL-1β, TNF-α, and IL-6 protein levels were quantified by ELISA, according to the manufacturers’ suggested protocol (R&D Systems, Abingdon, UK), using selective antibodies. Absorbance was read at 405 nm. The detection limit was < 3 pg/ml.

### Statistical analysis

All statistical analysis was performed using GraphPad Prism software (San Diego, CA, USA). Most comparisons between CTL and EL slices were made using an unpaired *t* test. Regarding PI uptake experiments and microglia cell body diameter measurements, statistical analysis was performed with one-way ANOVA, followed by Bonferroni’s comparison test. The number of independent cultures or cells (n) used in each assay is indicated in the legend of each figure. In all figures, data are presented as mean ± SEM. Values of *p* < 0.05 were considered to account for statistically significant differences.

## Results

### Slices maintained in Neurobasal A-serum-free medium exhibit interictal-like activity

As depicted in Fig. 1a, the organotypic slices used throughout this work contain the hippocampal complex, namely the dentate gyrus (DG) and the cornu ammonis areas (CA1 and CA3) of the hippocampus, as well as the entorhinal (EC) and perirhinal (PC) cortex. Indeed, clinical research and animal models of epilepsy have suggested that these cortical areas play an important role in seizure generation [50].

**Fig. 2:**
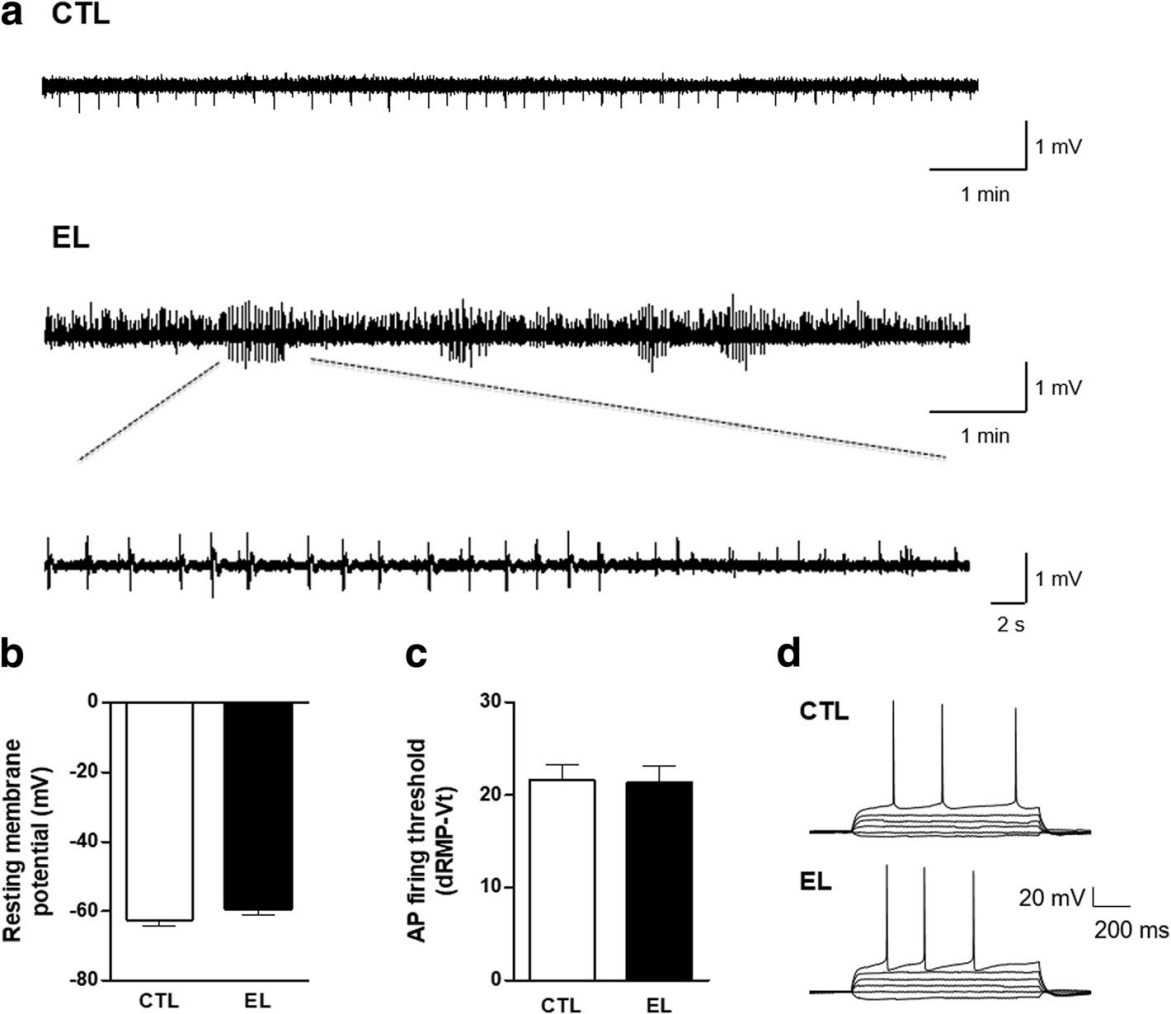
Electrophysiological properties of organotypic slices control (CTL) and with spontaneous epileptiform activity (EL). **a** Representative field potential recordings of spontaneous activity from CA3 area of CTL and EL organotypic slices. CTL slices depict a regular physiological activity, while EL slices exhibit epileptiform activity at 14 DIV. All vertical bars, 1 mV. **b** Resting membrane potential (RMP) of CA3 pyramidal neurons were measured immediately upon establishing whole-cell configuration and did not significantly differ between CTL and EL slices. **c** There were also no statistically significant changes between the action potential (AP) threshold measured from neurons in CTL and EL slices. **d** Representative tracings for each condition are shown. Data represent the mean ± SEM. *n* = 7–9 cells per condition, from 3 to 4 independent cultures, unpaired *t* test

Spontaneous activity of the slices was assessed at 14 DIV. CTL and EL slices were transferred to the interface chamber, and field potentials were recorded (40 min) under a constant superfusion with growth medium. Figure 2 shows representative field potential recordings of spontaneous activity obtained in CA3 area of CTL (Fig. 2a–CTL) and EL (Fig. 2a–EL) organotypic slices at 14 DIV. CTL slices depict a spontaneous typical physiological activity, while EL slices exhibit spontaneous interictal-like discharges, which resemble in vivo epilepsy, in accordance with what was previously described by others [42].

### CTL and EL slices have identical resting membrane potential and firing threshold

To evaluate the resting membrane potential of neurons, CTL and EL slices were transferred to the chamber of an upright microscope and RMP values of CA3 pyramidal cells were measured immediately upon establishing whole-cell configuration. We found no statistically significant differences between RMP measured in neurons from CTL slices when compared with neurons from EL slices (CTL − 62.57 ± 1.59 mV and EL − 59.25 ± 1.89 mV, *p* > 0.05, *t* test) (Fig. 2b).

Action potentials were then evoked under current-clamp mode by injecting current pulses (− 50 to + 300 pA, in 10 or 50 pA increments for 1000 ms) from an initial holding potential of − 70 mV. The threshold for AP generation was measured as the beginning of the upward rise of the AP when dV/dT exceeded 10 mV/ms. The delta between the RMP of each neuron and the threshold potential (Vt), dRMP-Vt, at which they first fired an AP was not significantly different (*p* > 0.05, *t* test) between CTL (dRMP-Vt 21.63 ± 1.63 mV) and EL (dRMP-Vt 21.40 ± 1.81 mV) slices (Fig. 2c, d).

### EL slices have increased doublecortin immunoreactivity

Over the last decade, changes in hippocampal neurogenesis have emerged as a hallmark of temporal lobe epilepsy (TLE) [14].

Doublecortin (DCX) is a microtubule-associated phosphoprotein, which is widely used as a marker for immature neurons derived from newly generated neural precursor cells and has been validated to assess changes in the level of neurogenesis [11].

Thus, an immunofluorescence protocol was performed to evaluate the DCX staining in the DG of CTL and EL slices at 14 DIV.

As can be observed in Additional file 2: Figure S2, DCX staining was increased in slices that display epileptiform activity. Indeed, DCX fluorescence intensity was found to be significantly higher (*p < 0.05, *t* test) in the DG of EL slices, thus suggesting increased neurogenesis.

### EL slices show higher αII-spectrin cleavage

Neuronal death is considered an important feature of epilepsy. To evaluate if neuronal death was increased in EL slices, we quantified αII-spectrin cleavage and caspase-3 activation by a western blot assay, carried out with protein extracts obtained from the hippocampal region of the slices. Calpain-mediated degradation of αII-spectrin results in the formation of two unique and highly stable SBDPs with a molecular weight of 145 and 150 kDa (SBDP145 and SBDP150), which can be further cleaved by caspase-3 yielding shorter fragments. The presence of the calpain-cleaved fragments is usually indicative of necrotic and excitotoxic neuronal death. On the other hand, caspase-3-mediated αII-spectrin cleavage leads to the formation of a fragment with 150 kDa (SBDP150), which is further degraded by caspase-3, producing the apoptotic-specific SBDP120, with 120 kDa [51].

**Fig. 3:**
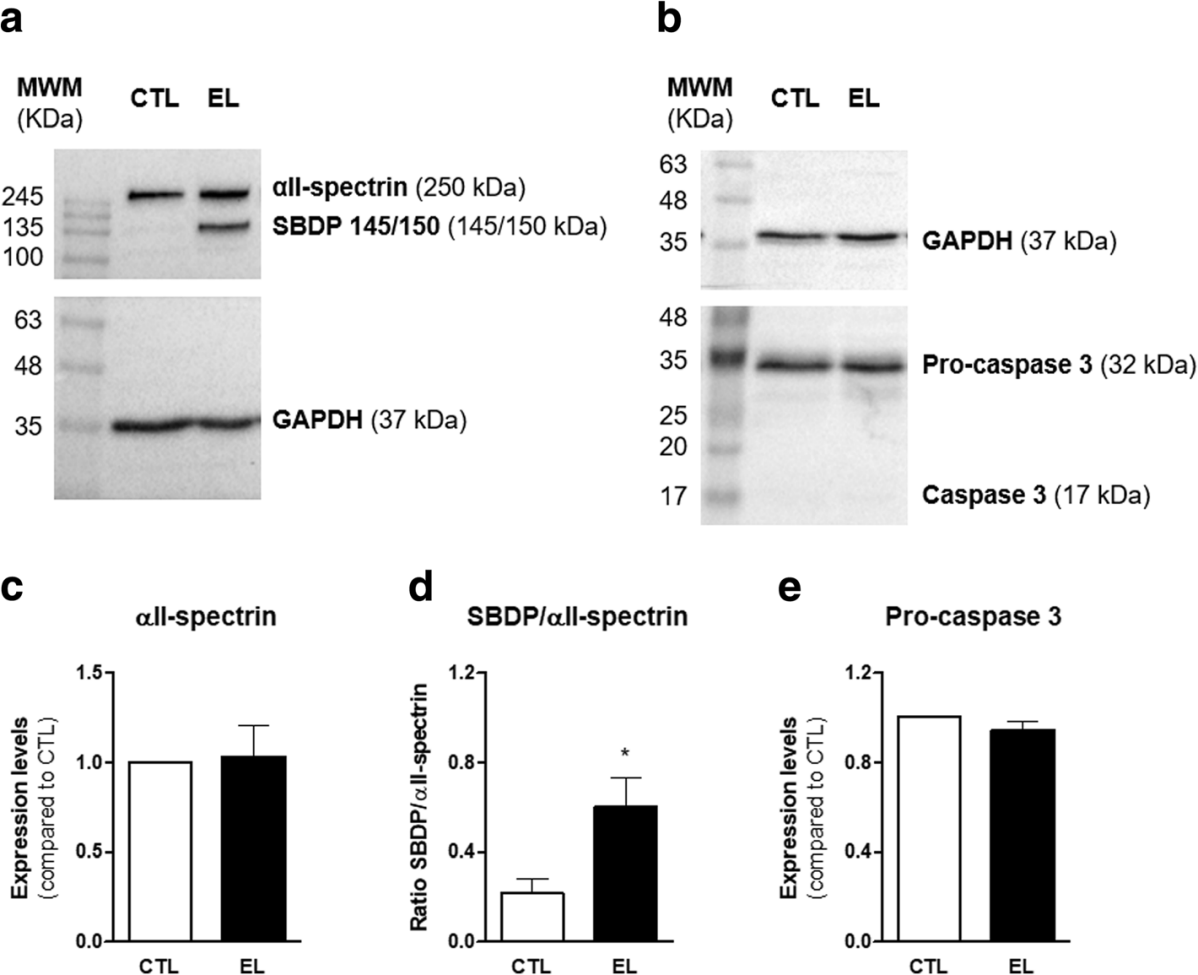
Western blot analysis of αII-spectrin cleavage and Pro-caspase 3 of organotypic slices control (CTL) and with spontaneous epileptiform activity (EL). **a**, **b** Representative immunoblots of αII-spectrin (250 kDa), SBDP 145/150 (145/150 kDa), Pro-caspase 3 (32 kDa), and GAPDH (37 kDa). **c**–**e** Densitometry analysis was performed with ImageJ software using GAPDH as internal control. **c** Full length αII-spectrin expression was similar between CTL and EL slices, while **d** the ratio SBDP 145/150 to αII-Spectrin was significantly upregulated in EL slices. **e** No differences were found in Pro-caspase 3 expression between CTL and EL slices. All values are mean ± SEM. *n* = 4, *p < 0.05, unpaired *t* test

Thus, an antibody which recognizes the full length αII-spectrin, as well as the calpain- and caspase-3-signature SBDPs, was used. As can be observed in Fig. 3a, this antibody identified two bands: a high molecular weight band, which corresponds to full length αII-spectrin (250 kDa), and a lower one for SBDPs. The expression of SBDP145 and SBDP150 was not possible to visualize separately (Fig. 3a). However, the apoptotic-specific SBDP120, which results from the caspase-3 cleavage of αII-spectrin, was absent from both conditions, indicating that the lower band recognized by αII-spectrin antibody corresponds exclusively to the calpain-signature SBDPs.

CTL and EL slices have a similar expression of full length αII-spectrin (Fig. 3c, *p* > 0.05, *t* test). The results also indicate that the ratio SBDP145/150 to αII-spectrin was significantly increased (Fig. 3d, *p < 0.05, *t* test) in EL slices, pointing to a higher αII-spectrin cleavage by calpain in these slices. Furthermore, the band for active caspase-3 (17 kDa) is absent from the immunoblot (Fig. 3b) and no differences are found in pro-caspase 3 expression (Fig. 3e, *p* > 0.05, *t* test). These results corroborate the sole detection of SBDP145/150 originated by calpain-mediated cleavage of αII-spectrin and point to an increased necrosis in EL slices.

### EL slices show increased PI uptake

To evaluate neuronal death across the different regions of the hippocampus, the PI uptake assay was carried out in CTL and EL slices at 14 DIV.

**Fig. 4:**
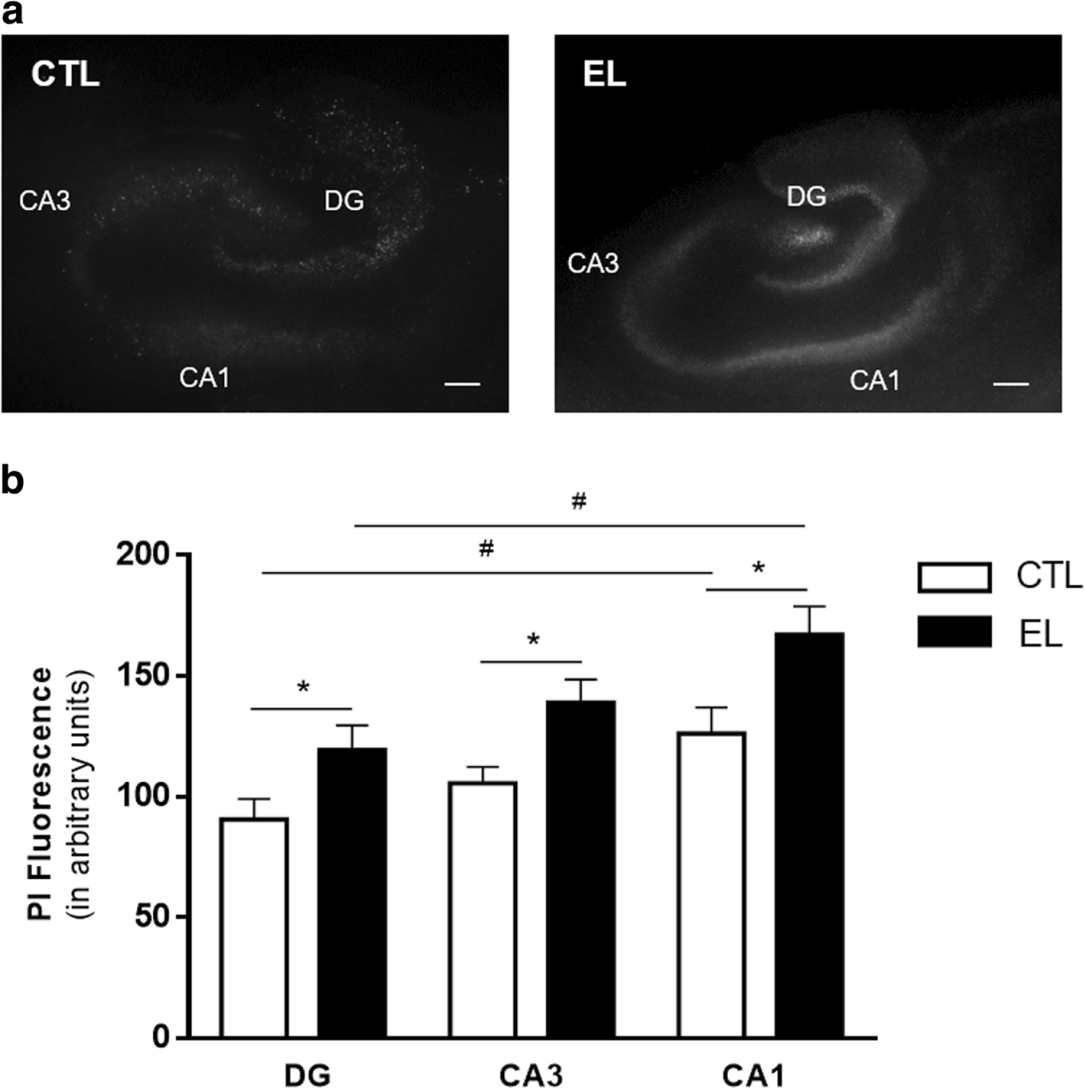
Propidium iodide uptake of organotypic slices control (CTL) and with spontaneous epileptiform activity (EL). **a** Representative fluorescence photomicrographs of PI uptake in CTL and EL slices. **b** Quantification of PI uptake by each hippocampal region, expressed in arbitrary units of fluorescence intensity, was performed with ImageJ software. In both conditions, the highest PI uptake was observed in CA1 pyramidal region. However, EL slices have a higher PI incorporation in all hippocampal areas when compared to CTL ones. All values are mean ± SEM. *n* = 17–29 slices per condition, from 3 to 4 independent cultures. Statistical tests were performed with one-way ANOVA, followed by Bonferroni’s comparison test, *p < 0.05, for comparisons within the same hippocampal area, ^#^*p* < 0.05, for comparisons within the same group, as depicted by the lines above the bars. Scale bar, 200 μm

The representative fluorescence photomicrographs of both groups of slices (Fig. 4a) suggested that PI incorporation was increased in EL slices. This observation was corroborated by the quantification of PI uptake in each hippocampal region, expressed in arbitrary units of fluorescence intensity (Fig. 4b). PI uptake in granular and pyramidal neurons of EL slices was significantly higher (*p < 0.05, one-way ANOVA) than that detected in the same regions of CTL slices. Also, in both groups of slices, CA1 pyramidal neurons show a significantly higher PI uptake than granular neurons (^#^*p* < 0.05, one-way ANOVA). Specifically, for DG, CTL 86.71 ± 8.016 vs EL 121.3 ± 10.51; for CA3, CTL 105.4 ± 6.920 vs EL 135.5 ± 9.846; and for CA1, CTL 130.1 ± 10.67 vs EL 169.8 ± 11.83.

### EL slices depict increased gliosis

Activation of astrocytes and microglia are considered a molecular hallmark of epilepsy, and recent findings point to a role of glial cells in the pathogenesis of this disorder [52, 53].

To investigate if gliosis was associated with epileptiform activity, two experimental approaches were used: a western blot assay to evaluate the overall expression of GFAP and Iba1 and an immunohistochemistry assay to evaluate morphological changes in astrocytes and microglia.

**Fig. 5:**
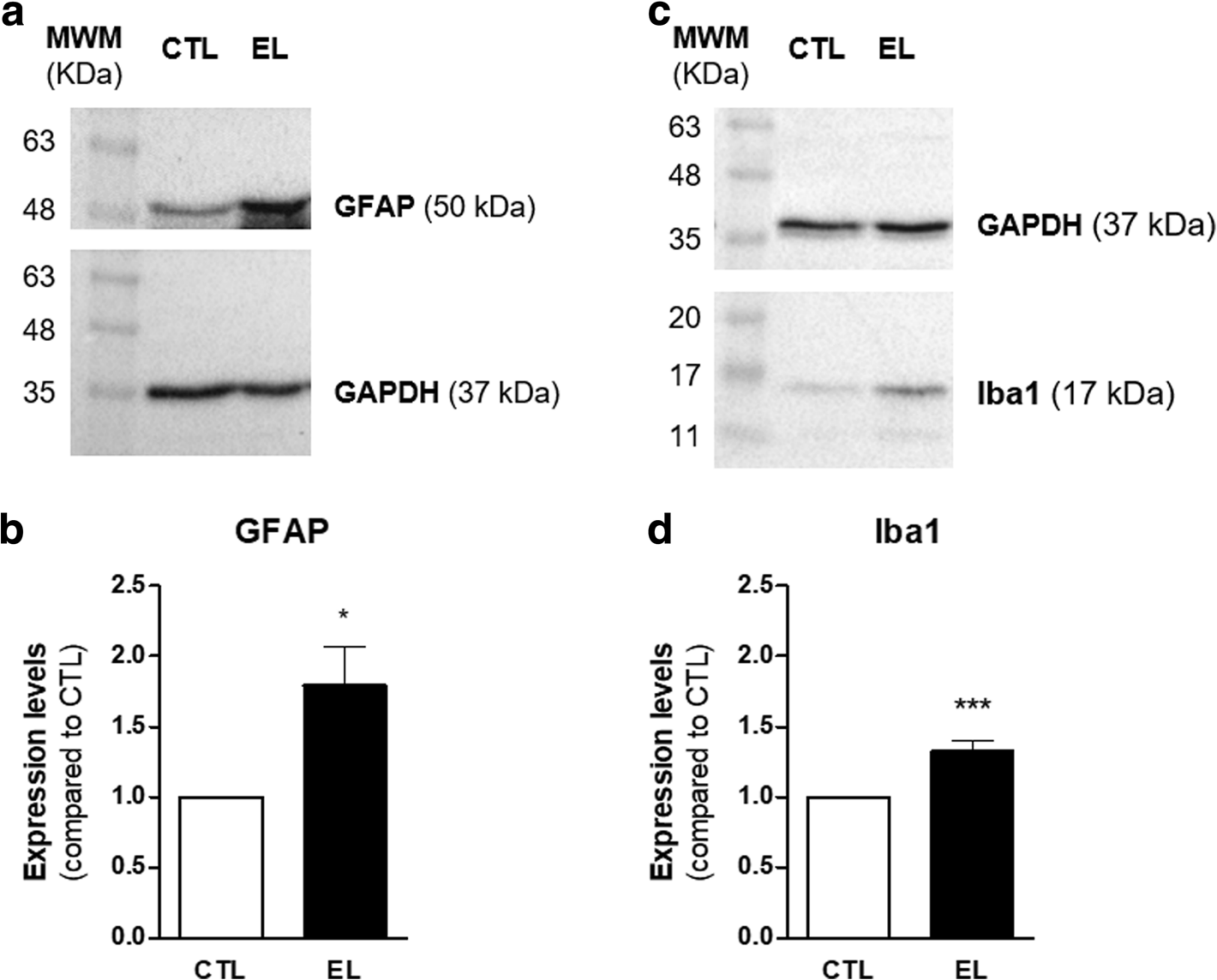
Western blot analysis of GFAP and Iba1 in organotypic slices control (CTL) and with spontaneous epileptiform activity (EL) at 14 DIV. **a**, **c** Representative immunoblots for GFAP (50 kDa), Iba1 (17 kDa), and GAPDH (37 kDa). **b**, **d** Densitometry analysis was performed with ImageJ software using GAPDH as internal control. GFAP and Iba1 expression are significantly upregulated in EL slices. All values are mean ± SEM. *n* = 4, *p < 0.05, ***p < 0.001, unpaired *t* test

The western blot was carried out with 14 DIV hippocampal lysates obtained from CTL and EL slices. The immunoblots (Fig. 5a, c) showed an increased expression of GFAP and Iba1 in EL slices. Indeed, the densitometry analysis (Fig. 5b) attested a significant increase in GFAP expression in EL slices (*p < 0.05, *t* test). Likewise, the increased Iba1 expression in EL slices, evident in the immunoblot (Fig. 5c), was supported by the densitometry analysis (Fig. 5d), which indicated that Iba1 expression was significantly higher (***p < 0.001, *t* test) in EL slices, when compared to CTL ones.

**Fig. 6:**
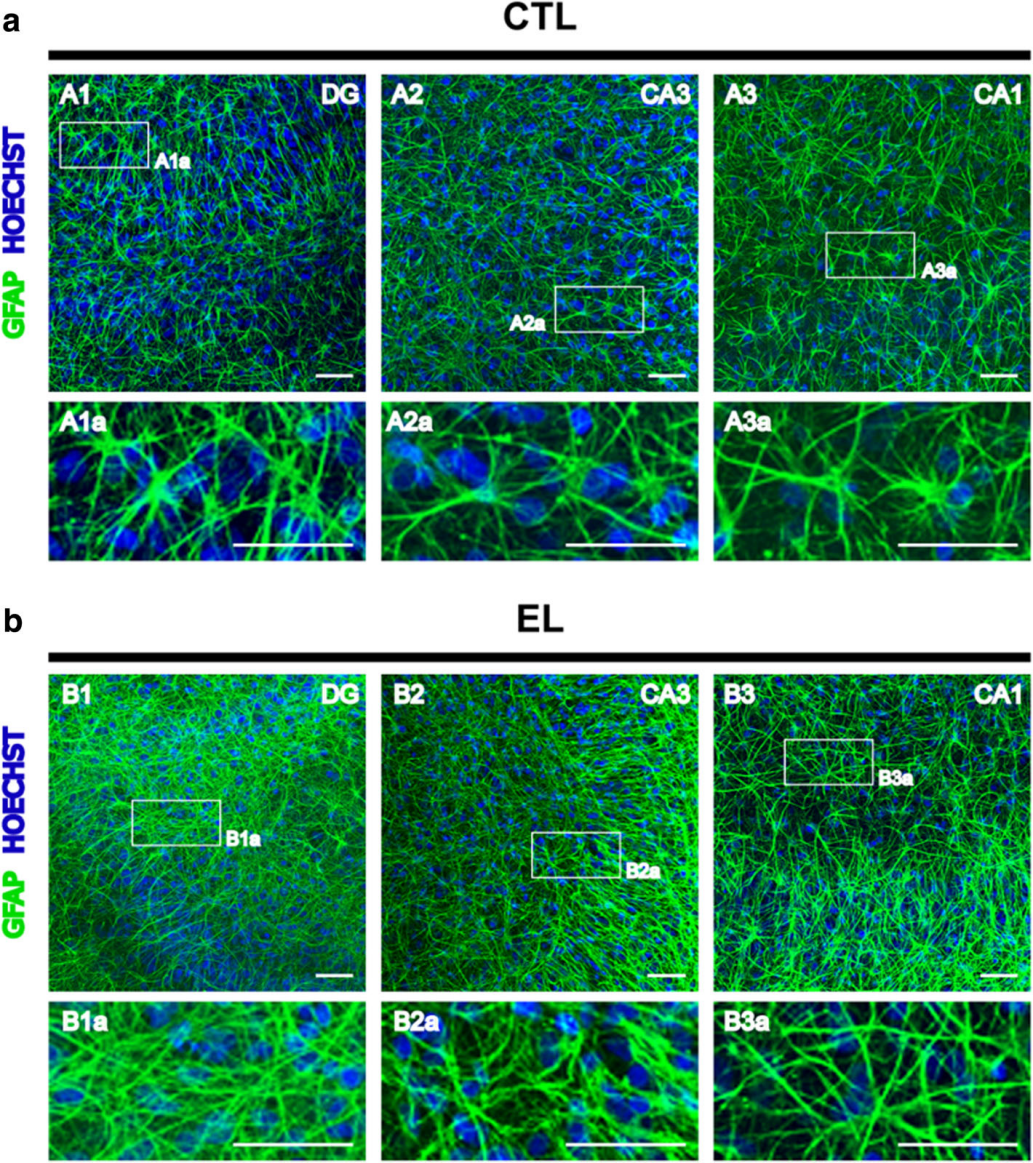
Representative morphology of astrocytes in organotypic slices control (CTL) and with spontaneous epileptiform activity (EL) at 14 DIV. Images of GFAP-stained astrocytes (green) and Hoechst-stained nuclei (blue) were acquired on a confocal laser microscope (Zeiss LSM 710) with a 20x objective. Magnified images of the dashed areas are shown. **a** In CTL slices, most astrocytes show thin processes with preservation of singular domains. **b** In EL slices, astrocytes have hypertrophic cell bodies, with extensive overlap of astrocytic processes and disruption of individual domains. Scale bars, 50 μm

**Fig. 7:**
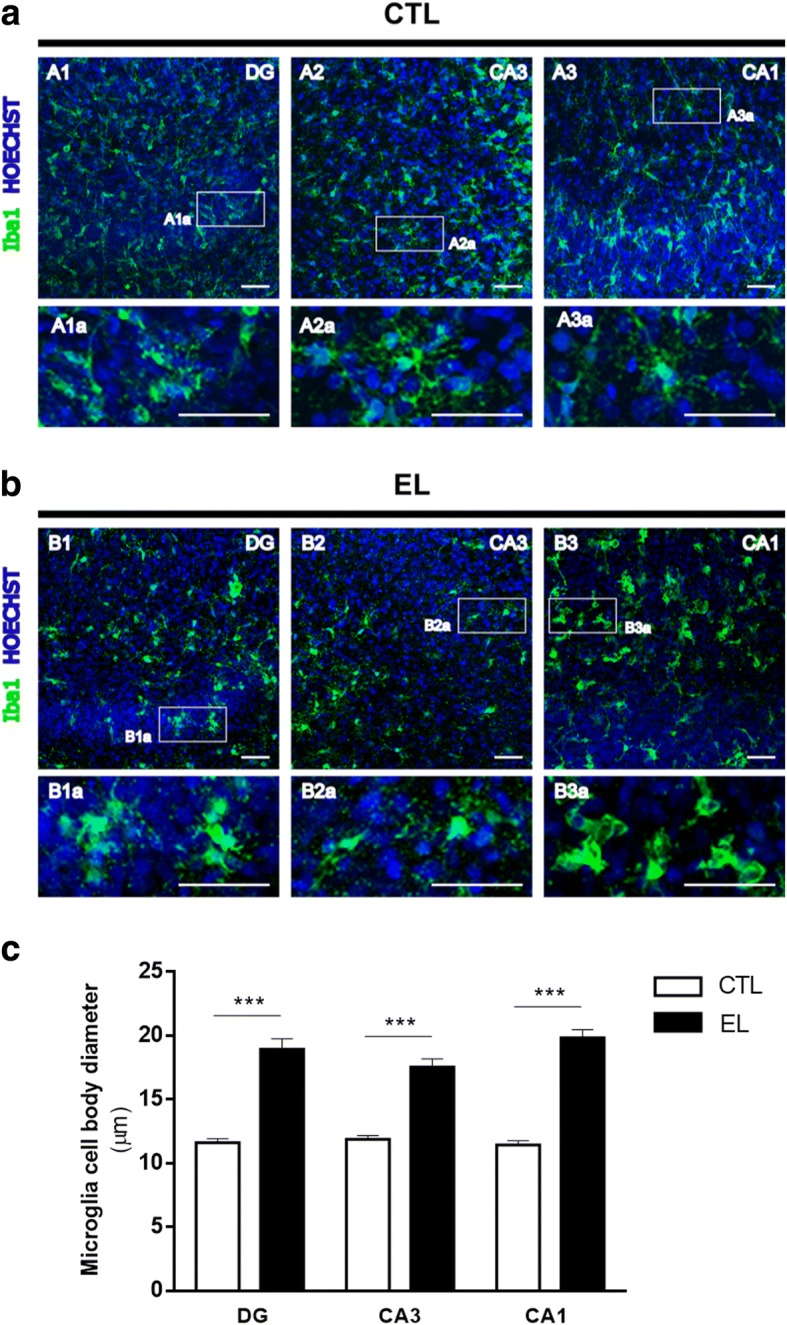
Representative morphology of microglia in organotypic slices control (CTL) and with spontaneous epileptiform activity (EL) at 14 DIV. Images of Iba1-stained microglia (green) and Hoechst-stained nuclei (blue) were acquired on a confocal laser microscope (Zeiss LSM 710) with a 20x objective. Magnified images of the dashed areas are shown. **a** Most microglia in CTL slices have a ramified morphology, characteristic of a resting state. **b** EL slices depict microglia with a large cell body and retracted processes, corroborating an activated “bushy-like” state. **c** The diameter of microglia cell body was quantified with ImageJ software. In EL slices, microglia show a significant increase in cell body diameter in all hippocampal areas. All values are mean ± SEM. *n* = 50–70 microglia cells per hippocampal area per condition, from 3 independent cultures, ***p < 0.001, one-way ANOVA, followed by Bonferroni’s comparison test. Scale bars, 50 μm

An immunofluorescence analysis was also performed in CTL and EL slices to assess the morphological features of astrocytes (Fig. 6) and microglia (Fig. 7) in the main hippocampal areas. At 14 DIV, slices were fixed with PFA and handled as described before.

In the DG and CA regions of CTL slices (Fig. 6a–A1, A2, A3), most GFAP-positive astrocytes have thin processes and display preservation of individual domains, which can be observed in the magnified panels, Fig. 6a–A1a, A2a, and A3a. However, some hypertrophic cell bodies can also be observed suggestive of a mildly activated state. This basal astrogliosis in organotypic slices has long been reported by others [54]. However, in EL slices (Fig. 6b), a moderate/reactive astrogliosis can be noticed in all regions of the hippocampus, which include the presence of tick and highly superimposed astrocytic processes, resulting in the disruption of individual domains. These features are visible in the magnified panels, Fig. 6b–B1a, B2a, and B3a.

Figure 7 shows the occurrence of microglia cells, identified by Iba1 expression, in all hippocampal regions of CTL and EL slices. In CTL slices (Fig. 7a), most microglia have a ramified morphology, characteristic of a resting state, as can be noticed in Fig. 7a–A1a, A2a, and A3a. In EL slices (Fig. 7b), microglia are more abundant in all regions of the hippocampus, which agrees with the western blot results described above. Furthermore, most microglia have larger cell bodies and have changed their morphology from ramified to bushy-like cells with fewer processes, as observable in Fig. 7b–B1a, B2a, and B3a. In comparison with CTL slices, microglia in EL slices have a significantly increased cell body diameter in all regions of the hippocampus (***p < 0.001, one-way ANOVA), as attested by the quantification depicted in Fig. 7c. Within each condition, no statistical difference was found between hippocampal areas (*p* > 0.05, one-way ANOVA). The mean microglia cell body diameter for CTL slices (DG 11.6 μm ± 0.32; CA3 11.9 μm ± 0.30; CA1 11.5 μm ± 0.31) is higher than 7.5 μm reported by others [55] in resting microglia. This points to a mixed population of resting and reactive microglia in CTL slices, which is in accordance with some basal microglia activation known to occur in organotypic slices [54]. However, in EL slices, the mean microglia cell body diameter increases approximately twofold in relation to CTL ones (DG 18.9 μm ± 0.82; CA3 17.6 μm ± 0.64; CA1 19.8 μm ± 0.63), supporting the qualitative morphological analysis.

Thus, the described morphological alterations depicted by astrocytes and microglia corroborate an increased astrogliosis and microgliosis in EL slices.

### Expression of proinflammatory cytokines is increased in EL slices

Recently, inflammatory-related events have been intensely discussed in the context of epilepsy and were proposed as potential therapeutic targets for new antiepileptic drugs [56, 57].

After showing the occurrence of gliosis in EL slices, it was pertinent to evaluate alterations in the expression of proinflammatory cytokines, namely IL-1β, TNF-α, and IL-6, which are expressed at very low levels in the normal brain, but undergo a fast increase in expression after the induction of seizures [58, 59].

Thus, the expression of these proinflammatory cytokines was evaluated at the mRNA and protein level in CTL and EL slices at 14 DIV.

**Fig. 8:**
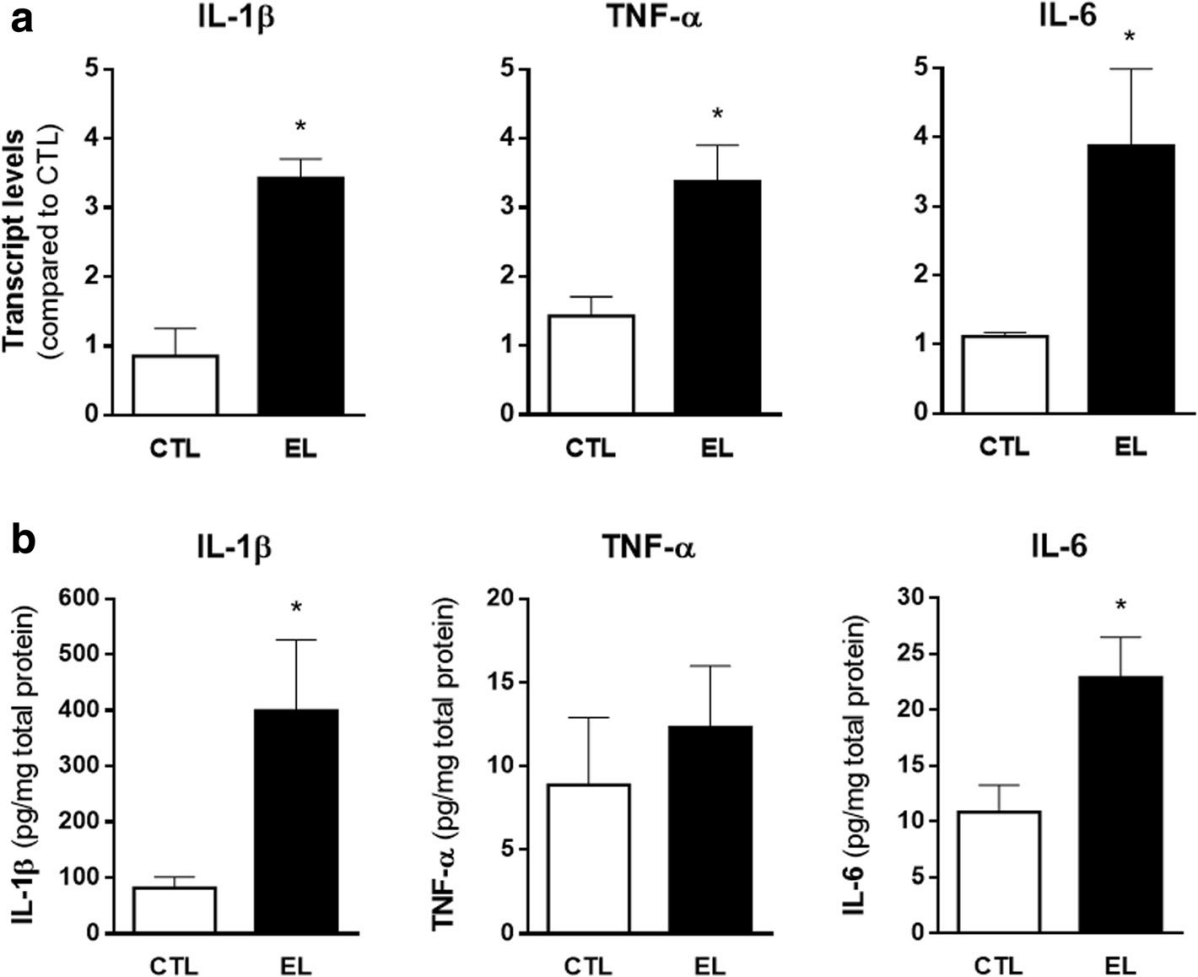
Expression of proinflammatory cytokines in organotypic slices control (CTL) and with spontaneous epileptiform activity (EL) at 14 DIV. **a** RT-qPCR analysis of IL-1β, TNF-α, and IL-6 shows a transcript upregulation for all cytokines in EL slices. **b** IL-1β and IL-6 protein concentration in tissue, assessed by ELISA, is also significantly increased in EL slices. All values are mean ± SEM. *n* = 3–4, *p < 0.05, unpaired *t* test

The transcript expression was evaluated by RT-qPCR, and the results are depicted in Fig. 8a. As can be observed, the mRNA expression of IL-1β, TNF-α, and IL-6 is significantly upregulated in EL slices (*p < 0.05, *t* test).

The protein concentration of these cytokines in the tissue, Fig. 8b, was calculated by an ELISA assay. At the time point evaluated, protein levels of IL-1β and IL-6 were found to be increased in EL slices (*p < 0.05, *t* test), but TNF-α change at the protein level did not reach statistical significance (*p* > 0.05, *t* test).

### NLRP3 expression is increased in EL slices

Recently, studies indicate a correlation between enhanced NLRP3 inflammasome expression and several neurological-related disorders [30]. NLRP3 activation leads to the processing of pro-IL-1β and pro-IL-18 by caspase-1, with the consequent increased production of the mature cytokines. Since IL-1β was upregulated in EL slices, it was relevant to evaluate if NLRP3 expression was altered in these slices.

**Fig. 9:**
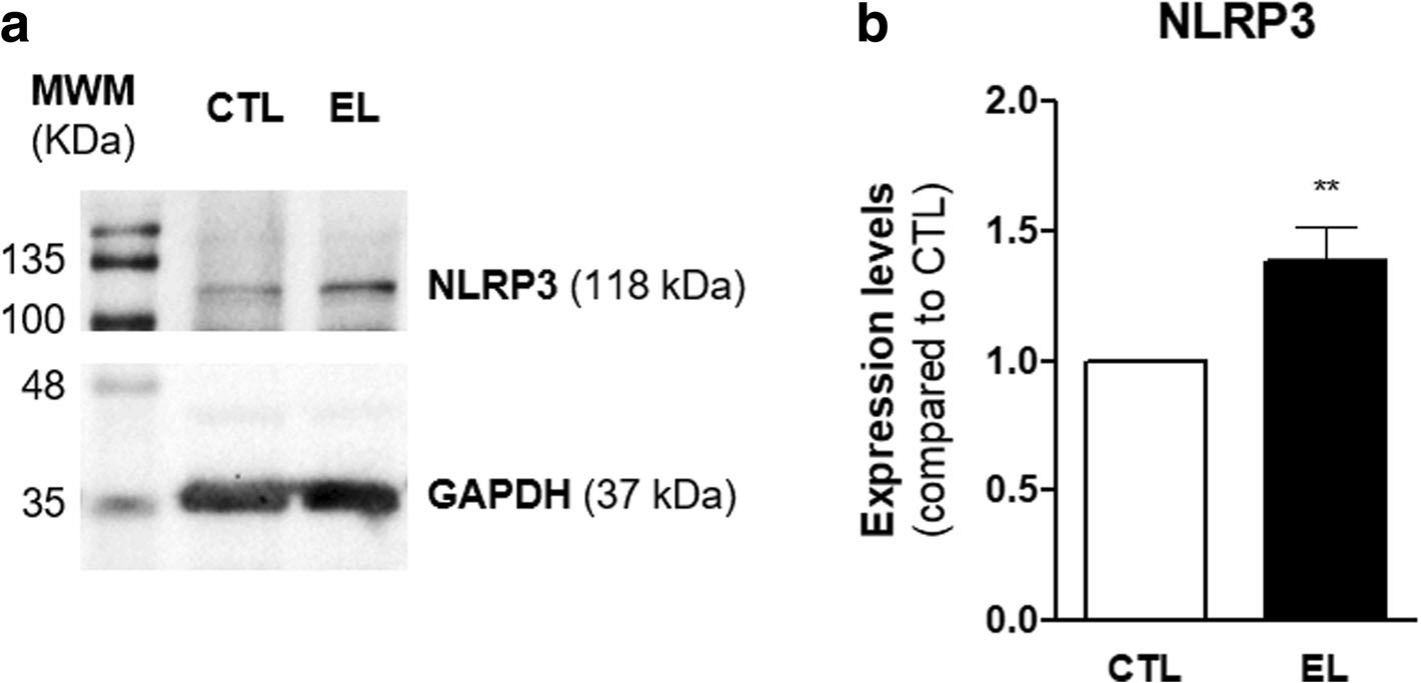
Protein levels of NLRP3 in organotypic slices control (CTL) and with spontaneous epileptiform activity (EL) at 14 DIV. **a** Representative immunoblot for NLRP3 (118 kDa) and GAPDH (37 kDa). **b** Densitometry analysis was performed with ImageJ software using GAPDH as internal control. NLRP3 expression is significantly upregulated in EL slices. All values are mean ± SEM. *n* = 4, **p < 0.01, unpaired *t* test

To evaluate the differential expression of NLRP3 inflammasome between CTL and EL slices, a western blot was carried out with 14 DIV hippocampal lysates. The immunoblot (Fig. 9a) shows that NLRP3 expression was increased in EL slices. This result was confirmed by the densitometric analysis (Fig. 9b), which indicated a statistical significant increase (*p < 0.05, *t* test) in NLRP3 expression in EL slices.

## Discussion

This work compares “healthy” organotypic slices with those displaying epileptiform activity, in terms of inflammatory features. We herein show that slices, which exhibit spontaneous epileptiform activity resembling in vivo epilepsy, also depict inflammatory features found in animal models of epilepsy and in TLE patients. We describe increased neuronal death, activation of astrocytes and microglia, and enhanced expression of proinflammatory cytokines, namely IL-1β, TNF-α, and IL-6. We also show that NLRP3 inflammasome expression is increased in slices depicting spontaneous activity. By recapitulating key inflammatory features of epilepsy, the proposed system is of value for mechanistic studies of this disease and can be used as a tool for evaluating potential therapeutic approaches.

Our work confirms the development of interictal-like spikes in combined entorhinal-hippocampus organotypic slices. It was already reported that epileptiform activity developed in organotypic slices later than 7 DIV, with primarily interictal-like spikes and bursts of activity at 14 to 17 DIV which preceded ictal-like discharges after 21 DIV [42]. These authors suggested that the reorganization of the local network caused by deafferentation and deefferentation occurring during tissue slicing could be the cause of spontaneous activity in organotypic hippocampal slice cultures. However, as we now demonstrate, slices maintained in a serum containing medium, herein denominated CTL slices, do not display spontaneous activity, precluding the possibility that slice preparation per se could be the only cause for spontaneous activity. In our work, CTL slices only depicted a regular activity (Fig. 2a–CTL), whereas slices progressively depleted of serum in the growth medium revealed interictal-like events (Fig. 2a–EL). Interictal spikes in electroencephalographic recordings from epilepsy patients are brief paroxysmal discharges observed between spontaneous recurrent seizures and have been regarded as markers for ongoing epileptogenesis [60]. Hence, according to our recordings, slices gradually deprived of serum in the growth medium mimic some aspects of epileptogenesis, while slices maintained in serum-based conditions do not. To our knowledge, such assessment was never carried out.

Serum-free and serum-replacement medium formulations have become essential for biotechnology industries. The elimination of animal components aims standardization, consistency, and reduced risks of contamination in cell culture processes. Furthermore, serum-free medium mimics the serum-free environment found in CNS parenchyma.

However, serum withdrawal from the maintenance medium may play a role in excitability since it affects hippocampal circuitry. It was reported that dendritic spine density and morphology in organotypic slices depend on the presence of serum in the culture media [61] and granule layer cell density was also decreased in the absence of serum [62]. It is therefore plausible to foresee that serum deprivation leads to persistent changes in the hippocampal circuitry that ultimately decrease the threshold for spikes. Furthermore, recent evidences have shown that serum deprivation increases reactive oxygen species (ROS) production [63]. Although ROS are removed by the antioxidant systems to maintain redox homeostasis, situations of excessive ROS production occur and have been implicated in many pathological disorders including cancer, aging, and neurological diseases [64]. Indeed, ROS generation was suggested to be one of the critical elements for NLRP3 activation [65, 66], one of the features that we observe in our system. Yet, the detailed mechanism through which serum deprivation contributes to epileptiform activity in organotypic slices was not matter of this work and is difficult to address due to the lack of detailed knowledge of the serum and culture media composition.

The hippocampus has become one of the most extensively studied area of the mammalian brain and is considered the most vulnerable brain region for damage. Indeed, its impaired function has been reported in many human brain diseases, such as hypoxia, ischemia, and epilepsy [36].

An association between neuronal death and epileptogenesis has been intensely debated [4]. This work assessed cell death by necrosis and apoptosis, using αII-spectrin cleavage, a major substrate of calpains and caspases, for that purpose. The calpain-mediated fragments of αII-spectrin cleavage (SBDP145/150) were found in slices with epileptiform activity (Fig. 3a), while the caspase-3-mediated apoptotic-specific fragment (SBDP120) was not. This result points to the occurrence of necrosis, which is associated with massive Na^+^ and Ca^2+^ influxes and thus with excitotoxicity. Nevertheless, a cross-talk between apoptosis and necrosis cascades [67], as well as caspase-independent cell death processes [68], is gaining recognition among the scientific community and cannot be discarded. Moreover, novel mechanisms of cell death [69, 70], beyond the traditional concepts of necrosis and apoptosis, including autophagy, phagoptosis, necroptosis, and pyroptosis, are nowadays being considered to have a role in epileptogenesis [71]. Indeed, the increased NLRP3 expression and activation observed in slices with epileptiform activity point to pyroptosis as one of those mechanisms. But only a complete evaluation of cell death throughout time in culture will fully clarify the cell death mechanisms relevant in this system.

Region-specific neuronal death, across the different regions of the hippocampus, was also explored by the PI uptake assay. PI is a nucleic acid dye that is excluded from viable cells, but can enter in dying or dead cells with compromised membranes and stain their nuclei [49]. We found some PI incorporation in all slices, regardless of culture condition, reflecting cell death as a consequence of the massive deafferentation and deefferentation which occurs during tissue slicing. Truly, although after a few DIV the development of synapses in organotypic slices matches synaptogenesis in vivo, it is known that the density of synaptic contacts decreases immediately after explantation [72]. Nevertheless, at 14 DIV, slices with epileptiform activity display a stronger incidence of PI uptake, and thus cell loss, than control slices (Fig. 4). Stronger PI staining was largely confined to the main neuronal cell layers of the hippocampus, such as DG granular layer and CA1 and CA3 pyramidal layers, suggesting that the dying cells were neurons and thus corroborating neuronal loss, as concluded by the results obtained from αII-spectrin cleavage.

An increased neurogenesis was found in the DG of the slices which display epileptiform activity, corroborating what is reported in many animal models of epilepsy [14]. However, the impact of the newly born granule cells in the progression of the epileptiform activity was not addressed in this study.

Alterations in glial cells near or at the site of a lesion, named as gliosis, constitute a pathological hallmark of damaged CNS tissue, but the way in which gliosis influences a specific neuropathological condition is determined by many factors, including type of disease, injury severity, time after insult, damage location, and cellular changes [53].

Many cellular and molecular processes have been associated with epileptogenesis. Neuroinflammation, which includes the synthesis/release of proinflammatory mediators by activated astrocytes and microglia is one of them. In fact, inflammatory events have been extensively discussed in the context of epilepsy and have been proposed as having a role in the pathophysiology of this neurological disorder [73, 74]. Nowadays, brain inflammation is pointed out as a potential biomarker in epilepsy [75]. In our system, slices with epileptiform activity show noticeable signs of inflammation when compared to control slices, specifically activation of astrocytes and microglia, increased expression of proinflammatory cytokines, namely IL-1β, and NLRP3 overexpression and activation.

Our results clearly point to the loss of individual domains and extensive overlap of astrocytic processes in slices with epileptiform activity (Fig. 6). It is important to point out that the understanding about the functions and effects of reactive astrogliosis, and their impact on neural function, are still at an early stage. Nowadays, researchers still have a prevalent negative view of reactive astrogliosis and ultimately of glial scar formation, but new lines of evidence point towards its beneficial functions, particularly concerning neural protection and repair, and regulation of CNS inflammation [20]. In this paradoxical view, reactive astrocytes act not only to activate inflammation, at early times after insults, but also to form cell migration barriers that delineate areas of intense inflammation, thereby restricting the spread of inflammatory cells into nearby healthy tissue [76].

Together with reactive astrogliosis, increased microglia proliferation and microglia morphological changes were also observed in slices with epileptiform activity (Fig. 7). In contrast to the resting microglia state observed in control slices, microglia exhibit an activated “bushy-like” appearance in slices with epileptiform activity. We anticipated the occurrence of some amoeboid fully functional phagocytic microglial cell to clear up the cellular debris derived from neuronal death, but amoeboid microglia was not detected at the time point under evaluation.

Interestingly, this work has shown that slices with epileptiform activity demonstrated an increased mRNA expression of proinflammatory cytokines, namely IL-1β, TNF-α, and IL-6 (Fig. 8). Indeed, these cytokines exhibit a low expression level in healthy brain, but their messenger RNA (mRNA) and protein levels were reported to be rapidly increased after the induction of seizures [59]. At the protein level, only IL-1β and IL-6 show an increase in EL slices. TNF-α protein levels remain unchanged between CTL and EL slices, which can be attributed to regulatory processes occurring after TNF-α mRNA production [77] or to the time point under evaluation. In face of the gliosis herein detected, we anticipate that reactive astrocytes and activated microglia are the major contributors for the increased expression of proinflammatory cytokines, as described by others [58, 78].

Furthermore, NLRP3 overexpression in slices with epileptiform activity, together with the increased production of IL-1β, points to a role for NLRP3 inflammasome signaling in epileptogenesis and corroborates that NLRP3 may represent a potential therapeutic target for the treatment of epilepsy, as suggested [31]. Indeed, NLRP3 is nowadays considered a promising therapeutic target for the treatment of neuroinflammation-associated neurological diseases [79].

It is worthwhile to notice that organotypic slice cultures have become a powerful tool to study physiological and pharmacological properties of tissues, since they reproduce the in vivo environment, maintaining the cytoarchitecture of the original tissue. Also, they are relatively inexpensive, markedly reduce the number of animals, and are easy to prepare with no requirements for lengthy animal surgery nor arduous monitorization of physiological parameters, complying with the 3Rs (reduce, refine, replace). In OHSC, the effects of compounds and drugs can be examined without concern about their ability to pass through the BBB and most molecular biology techniques can be applied. However, some drawbacks are still pointed to these types of cultures, namely RNA/protein extraction from few slices, cell morphology assessment with 5–7 cell layers, and the variability in PI staining.

Organotypic slice cultures are established in the study of brain disorders [80]. The most explored brain area in organotypic slice cultures is the hippocampus. Hippocampal organotypic slice cultures are considered adequate to explore the basic mechanisms of epileptogenesis [40, 41], to study stroke and trauma brain injury [81], to study beta-amyloid toxicity, as a model for Alzheimer’s disease, and other neurodegenerative diseases [40], and are described to be suitable for neuroprotection studies [39]. Ventral mesencephalon and striatum, as well as cortex-corpus callosum-striatum-SN, organotypic slices, were explored to study Parkinson’s disease [80, 82], and organotypic cerebellar slice cultures, which mimic many aspects of axon myelination and cerebellar functions, appear to be the best alternative to in vivo experiments and the most commonly used model for investigating novel therapeutic strategies in multiple sclerosis [83]. It was also reported that organotypic brain slices are an excellent tool to address the therapeutic potential of stem cells, since they allow to study the effect of cell grafts, alone or combined with biomaterials, and to assess the responses of endogenous and implanted cells, as well as their interaction [82]. Nevertheless, although organotypic slice cultures are considered an excellent screening tool, they cannot replace in vivo models, which remain indispensable to evaluate the functional outcome of any therapeutic strategy.

The system herein described, which uses entorhinal cortex-hippocampus organotypic slices, opens a new window to explore neuroinflammation as a cause/consequence of epileptogenesis and reinforces the value of organotypic brain slice cultures as a platform to study brain function.

## Conclusions

In conclusion, this system is an excellent tool for monitoring epileptogenesis and the dynamics of spontaneous activity. It also mimics the inflammatory events associated with in vivo epilepsy and allows to interfere pharmacologically with inflammatory pathways, such as the NLRP3 inflammasome signaling, before and after the onset of epileptic-like events, prompting the screening of potential targets for antiepileptic drugs.

## Additional files

Additional file 1: **Figure S1.** Melting curves obtained by RT-qPCR of IL-1β, TNF-α, IL-6 and GAPDH transcripts. Y axis represents the first derivate of raw fluorescence and X axis corresponds to temperature. Each curve has a single melting peak, which indicates that a single PCR product is being amplified. (TIF 46 kb)

Additional file 2: **Figure S2.** Neuronal differentiation in the DG of organotypic slices control (CTL) and with spontaneous epileptiform activity (EL) at 14 DIV. **a** Images of Doublecortin (DCX) stained immature neurons (red) and Hoechst stained nuclei (blue) were acquired on a confocal laser microscope (Zeiss LSM 710) with a 20x objective. **b** Doublecortin fluorescence intensity, quantified with ImageJ software, is significantly higher in EL slices. All values are mean ± SEM. *n* = 8 slices per condition, from 3 independent cultures, *p < 0.05, unpaired t-test. Scale bars, 50 μm. (TIF 352 kb)

## Abbreviations

aCSF: Artificial cerebrospinal fluid
AEDs: Antiepileptic drugs
AP: Action potential
BBB: Blood-brain barrier
BSA: Bovine serum albumin
CA: Cornus ammonis
Ct: Threshold cycle
CTL: Control slices
DCX: Doublecortin
DG: Dentate gyrus
DIV: Days in vitro
EC: Entorhinal cortex
EL: Slices with spontaneous epileptiform activity
GAPDH: Glyceraldehyde-3-phosphate dehydrogenase
GFAP: Glial fibrillary acidic protein
HBSS: Hanks’ balanced salt solution
HS: Horse serum
Iba1: Ionized calcium-binding adapter molecule 1
IL-1β: Interleukin-1β
IL-6: Interleukin-6
mRNA: Messenger RNA
MWM: Molecular weight marker
NLRP3 inflammasome: Nucleotide-binding, leucine-rich repeat, pyrin domain containing 3
OHSC: Organotypic hippocampal slice cultures
PBS: Phosphate-buffered saline
PC: Perirhinal cortex
PFA: Paraformaldehyde
PI: Propidium iodide
PVDF: Polyvinylidene difluoride
RMP: Resting membrane potential
ROS: Reactive oxygen species
RT-qPCR: Quantitative real-time PCR
SBDP: Spectrin breakdown products
SDS: Sodium dodecyl sulfate
TLE: Temporal lobe epilepsy
TNF-α: Tumor necrosis factor-α
