# Supplementary figures and images for: Ex vivo model of epilepsy in organotypic slices—a new tool for drug screening
Source: J Neuroinflammation. 2018 Jul 11;15:203. doi: 10.1186/s12974-018-1225-2 (PMC6042335; doi:10.1186/s12974-018-1225-2)

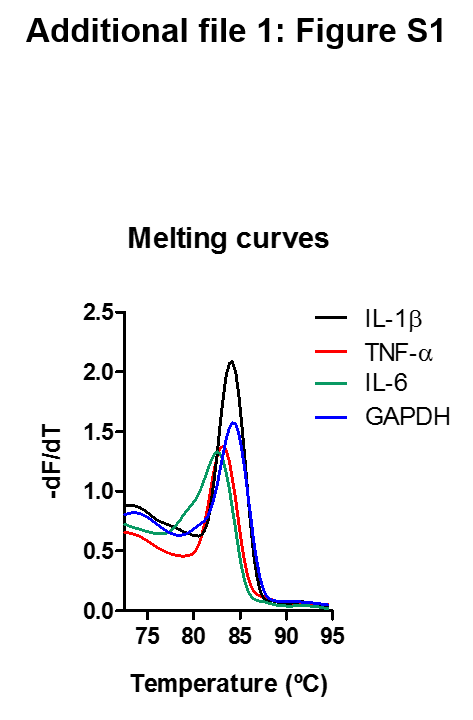

Supplement: Supplementary file 1 — Figure S1. Melting curves obtained by RT-qPCR of IL-1β, TNF-α, IL-6 and GAPDH transcripts. Y axis represents the first derivate of raw fluorescence and X axis corresponds to temperature. Each curve has a single melting peak, which indicates that a single PCR product is being amplified. (TIF 46 kb) [file 12974_2018_1225_MOESM1_ESM.tif]

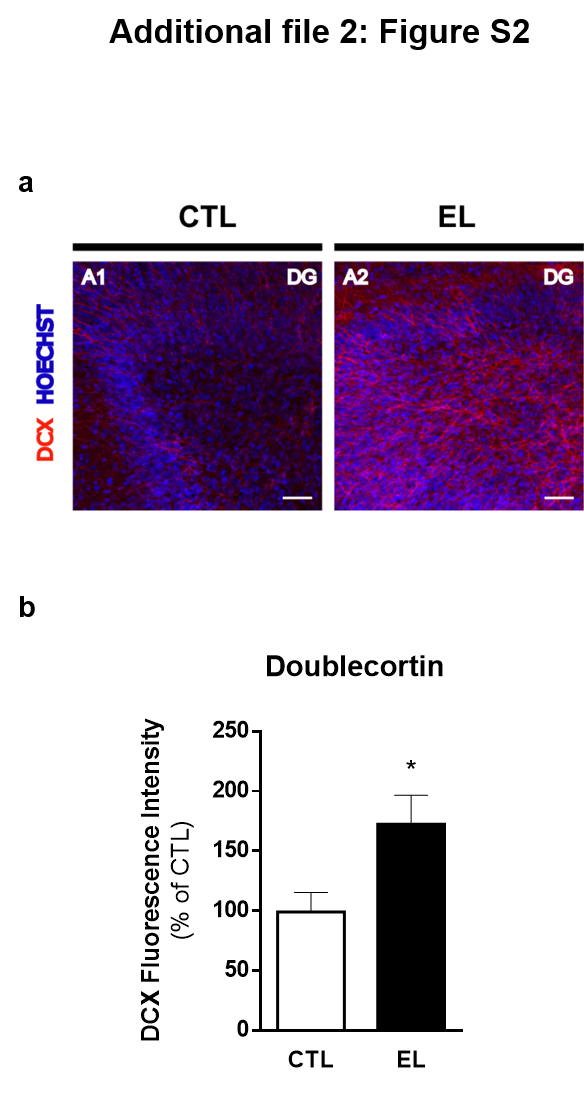

Supplement: Supplementary file 2 — Figure S2. Neuronal differentiation in the DG of organotypic slices control (CTL) and with spontaneous epileptiform activity (EL) at 14 DIV. a Images of Doublecortin (DCX) stained immature neurons (red) and Hoechst stained nuclei (blue) were acquired on a confocal laser microscope (Zeiss LSM 710) with a 20x objective. b Doublecortin fluorescence intensity, quantified with ImageJ software, is significantly higher in EL slices. All values are mean ± SEM. n = 8 slices per condition, from 3 independent cultures, *p < 0.05, unpaired t-test. Scale bars, 50 μm. (TIF 352 kb) [file 12974_2018_1225_MOESM2_ESM.tif]
